# Comparing the use of reverse total shoulder arthroplasty for glenohumeral osteoarthritis, irreparable rotator cuff tears, and rotator cuff tears with arthritis: a 2-year clinical outcome analysis using the Enovis AltiVate reverse

**DOI:** 10.1016/j.jsea.2026.100080

**Published:** 2026-08-11

**Authors:** Matthew R. Rohl, Christopher Miller, Taylor J. Manes, Tyler Hasbrook, Molly Moor, Scott P. Stephens, Brian L. Badman

**Affiliations:** a Ohio University Heritage College of Osteopathic Medicine, Dublin, OH, USA; b Department of Orthopedic Surgery, Ohio Health Doctors Hospital, Columbus, OH, USA; c Central Indiana Orthopedics, Fishers, IN, USA; d OrthoNeuro, Shoulder Division, New Albany, OH, USA

**Keywords:** Reverse total shoulder arthroplasty, Patient-reported outcomes, Irreparable rotator cuff tear, Glenohumeral osteoarthritis, Rotator cuff tear, Subscapularis repair

## Abstract

**Background:**

The purpose of this study was to compare outcomes of patients undergoing reverse total shoulder arthroplasty (rTSA) for rotator cuff tears with arthritis, irreparable rotator cuff tears without arthritis, and primary glenohumeral osteoarthritis.

**Methods:**

A retrospective review of prospectively collected data was performed of patients undergoing primary rTSA using the AltiVate Reverse (Enovis; Austin, TX) by a single surgeon from 2021 to 2023. Patients were categorized as irreparable rotator cuff tear without arthritis, rotator cuff tear with arthritis, or primary glenohumeral osteoarthritis. Outcomes included American Shoulder and Elbow Surgeons (ASES) score, Simple Shoulder Test (SST), visual analog scale (VAS) pain score, and range of motion, collected pre-operatively and through 2-year follow-up. Statistical analysis included Kruskal-Wallis, Chi-square, Mann-Whitney *U*, and Fisher exact tests (*P* < .05).

**Results:**

A total of 219 patients (221 shoulders) were included. All groups demonstrated significant improvements in ASES, SST, VAS, and range of motion at final follow-up (*P* < .05). Post-operative VAS, ASES, and SST scores were comparable between groups (all *P* > .05). External rotation at 2 years was greater in the osteoarthritis cohort compared with rotator cuff tear with arthritis (65° vs. 50°, *P* = .014). Overall complication rates were low and similar between groups (*P* = .686). Subscapularis repair was associated with a higher complication rate compared with no repair (22.7% vs. 11.8%, *P* = .043).

**Conclusion:**

rTSA provides comparable clinical outcomes across indications of irreparable rotator cuff tear, rotator cuff tear arthropathy, and primary osteoarthritis at 2 years, supporting its broad applicability.

Reverse total shoulder arthroplasty (rTSA) was originally designed over 3 decades ago primarily for treating patients with rotator cuff tear arthropathy.^4^^,^^8^^,^^12^^,^^19^ Since then, rTSA has become increasingly used for a variety of shoulder pathologies, including primary glenohumeral osteoarthritis, massive rotator cuff tears, proximal humerus fractures, rheumatoid arthritis affecting the glenohumeral joint, and revision surgeries.^3^^,^^12^^,^^17^ With these expanded indications, the use of rTSA has continued to rise. Mayfield et al^15^ found that primary rTSA accounted for up to 70% of primary shoulder arthroplasty in 2020 in the United States, a marked increase from 55% in 2016. A similar study found that the incidence of rTSA increased from 7.3 cases to 19.3 cases per 100,000 persons from 2012 to 2017.^1^^,^^16^ Over this time, rTSA has demonstrated reliable long-term clinical and functional outcomes across multiple cohorts.^3^^,^^22^

Despite the widespread adoption of rTSA for multiple diagnoses, it remains uncertain whether post-operative outcomes are consistent across the various indications. Much of the current evidence suggests that pre-operative diagnosis can influence both functional recovery and complication rates.^9^^,^^12^^,^^16^^,^^19^^,^^20^^,^^24^ Systematic reviews and cohort studies have previously shown that patients with primary glenohumeral osteoarthritis tend to have the most favorable outcomes.^9^^,^^19^^,^^20^^,^^24^ Patients undergoing rTSA for revision surgery or rheumatoid arthritis may experience higher complication rates and less predictable improvements, largely attributed to compromised glenoid bone stock and soft tissue disruption from prior surgery.^8^^,^^12^^,^^18^ Many prior studies on rTSA outcomes have focused on a single pre-operative diagnosis, compared only 2 indications at a time, or have compared rTSA to anatomic total shoulder arthroplasty (aTSA). Systematic reviews have also referenced limitations due to the various implants, surgical techniques, and rehabilitation protocols used across different studies.^12^ With the expanded indications, it remains important to determine whether comparable outcomes are achieved across the more commonly used indications which can be critical for patient education, shared decision-making, and surgical planning.

The present study aimed to evaluate and compare 2-year post-operative clinical and functional results of rTSA in patients treated for 3 different common indications: irreparable rotator cuff tear without arthritis, rotator cuff tear with arthritis, and primary osteoarthritis. All procedures were performed by a single fellowship-trained shoulder and elbow surgeon at a single hospital using a consistent surgical approach, implant design, and follow-up protocol. We hypothesized that despite differences in pre-operative shoulder pathology, there would be no significant differences in post-operative outcomes among the groups.

## Materials and methods

### Study design

A retrospective review of prospectively collected data from consecutive patients who underwent primary rTSA at a single institution between 2021 and 2023 by a single fellowship-trained shoulder and elbow orthopedic surgeon. Institutional review board approval was secured before starting this study. Patients aged 18 years or older who underwent rTSA for 1 of 3 indications: irreparable rotator cuff tear without arthritis, rotator cuff tear with arthritis, or primary glenohumeral osteoarthritis were included in this study. All patients had a minimum of two years of clinical and radiographic follow-up. The indication was made based on standard radiographs, magnetic resonance imaging or computed tomography scans, and intraoperative findings by the senior surgeon (B.L.B.). Patients with rheumatoid arthritis, prior infection or tumor, less than two years of follow-up, acute fractures, off-label use of hybrid implants, and those who underwent revision rTSA were excluded.

All procedures were performed using a deltopectoral approach. The implant system used across the cohort was the Altivate Reverse (Enovis; Austin, Texas). Glenoid components were implanted with an inferior tilt, and fixation was achieved using a monoblock baseplate with a central compression screw and peripheral locking screws. Subscapularis repair was performed selectively at the discretion of the surgeon depending on tendon quality and tension. The majority of subscapularis tendons were managed using a subscapularis peel technique, with lesser tuberosity osteotomy used for a minor subset of patients.

Post-operatively, patients were immobilized in a sling for six weeks. Passive range of motion (ROM) exercises were initiated immediately, followed by active-assisted and active-unassisted motion at 6 weeks. Strengthening exercises commenced at 10 weeks, progressing based upon each patient's tolerance, and all patients followed a standardized outpatient rehabilitation protocol supervised by certified physical therapists.

### Outcome measurements

Demographic data collected included age, sex, body mass index (BMI), sidedness, dominant arm, and comorbidities such as osteopenia, chronic obstructive pulmonary disease (COPD), autoimmune disease, diabetes mellitus, obesity, smoking status, and anticoagulant use. Pre-operative and post-operative clinical evaluations were carried out at 2 weeks, 6 weeks, 3 months, 6 months, 1 year, and 2 years by the treating surgeon. Patient-reported outcome measurements, including American Shoulder and Elbow Surgeons (ASES) score, Simple Shoulder Test (SST), and visual analog scale (VAS) for pain, were used for clinical evaluation. Active ROM, including forward elevation, abduction, external rotation, and internal rotation, was assessed as well. Internal rotation was assessed on a scale of 1-10 based on anatomical landmarks, with higher values indicating a greater ROM (2 points = buttock/greater trochanter, 4 points = L4-sacrum, 6 points = L1-3, 8 points = T8-12, 10 points = T1-7). ROM measurements were obtained with a standard goniometer. The pre-operative radiographic measurements collected by the senior surgeon included the glenoid lateralization angle, distalization angle, and Walch classification. Complications, including acromion and/or scapula fracture, humerus fracture distal to the stem, greater tuberosity fracture, infection, dislocation, periprosthetic fracture, subscapularis tear, acromial stress reaction, and component failure, were prospectively collected and retrospectively reviewed. A sub-group analysis was performed based on whether the subscapularis tendon was repaired at the time of rTSA.

### Statistical analysis

Data were checked for normality, and appropriate descriptive statistics, including frequencies with percentages, means with standard deviations, and medians with interquartile ranges, were computed for all variables. The minimal clinically important difference (MCID) for rTSA was previously defined by Simovitch et al^23^ and thresholds of −1.5, 13.9, and 17.5 were used for VAS, ASES, and SST scores, respectively. For ROM, thresholds of 16° for forward flexion, 13° for abduction, 4° for external rotation, and 0.2 for internal rotation were used. Kruskal-Wallis tests with Dunn post hoc comparisons, Chi-square, and Fisher exact tests were performed to compare variables with respect to diagnosis. Mann-Whitney *U* tests, Chi-square, and Fisher exact tests were used to compare variables by intervention. Data were analyzed SPSS (Version 29; IBM Corp., Armonk, NY, USA). All tests were two-tailed, and a *P* value <.05 was used to determine significance.

## Results

### Demographic, pre-operative, and perioperative data

A total of 219 patients (221 shoulders) met the inclusion criteria for this study. Patients were stratified by indication group: rotator cuff tear without arthritis (n = 32), rotator cuff tear with arthritis (n = 150), and glenohumeral osteoarthritis (n = 39). The median age of the cohort was 72 years (interquartile range, 66–77 years) and the mean BMI was 29.4 ± 6.1 kg/m^2^. The cohort was predominantly female (57%) and right sided (56%). Demographics were comparable between the 3 groups with respect to age (*P* = .139), BMI (*P* = .330), sex (*P* = .687), surgical side (*P* = .360), dominant arm involvement (*P* = .394), or comorbidities including osteopenia, COPD, autoimmune disease, diabetes mellitus, smoking status, and anticoagulant use (*P* > .05). Sample sizes varied slightly across outcome measures and time points due to missing data. Demographic data can be seen in Table I.

**Table I:** Pre-operative characteristics by diagnosis among patients with 2-y follow-up (N = 221).

Variable	Cuff tear without arthritis (N = 32) median (IQR), mean ± SD, or n (%)	Cuff tear with arthritis (N = 150) median (IQR), mean ± SD, or n (%)	Osteoarthritis (N = 39) median (IQR), mean ± SD, or n (%)	*P* value
Age (years)	67.9 (62.6-76.5)	72.7 (66.5-77.5)	72.8 (66.1-77.0)	.139
BMI (kg/m^2^)	31.8 (23.9-36.6)	28.4 (25.3-35.0)	31.0 (26.5-40.8)	.330
Female sex	16 (50.0)	87 (58.0)	23 (59.0)	.687
Surgical side				.360
Right	21 (65.6)	84 (56.0)	19 (48.7)	
Left	11 (34.4)	66 (44.0)	20 (51.3)	
Surgery on dominant arm	15 (65.2)	54 (56.1)	15 (46.9)	.394
Prior shoulder surgery	11 (34.4)	32 (21.5)	7 (18.9)	.236
Comorbidities				
Osteopenia	6 (18.8)	44 (29.5)	6 (15.8)	.141
COPD	3 (9.4)	6 (2.8)	1 (2.7)	.425
Autoimmune disorder	3 (9.4)	9 (6.0)	3 (8.1)	.656
Diabetes	4 (17.4)	17 (9.6)	5 (15.6)	.847
Current smoker	2 (8.7)	3 (2.5)	0 (0)	.170
Anticoagulant	5 (21.7)	28 (22.8)	10 (31.3)	.582
Walch classification				<.001
A1	31 (96.9)	121 (81.2)	21 (53.8)	
A2	0 (0)	6 (4.0)	3 (7.7)	
B1	0 (0)	1 (0.7)	0 (0)	
B2	1 (3.1)	6 (4.0)	4 (10.3)	
B3	0 (0)	15 (10.1)	9 (23.0)	
C	0 (0)	0 (0)	1 (2.6)	
D	0 (0)	0 (0)	1 (2.6)	
Pre-operative lateralization angle	106.0 (97.8-108.8)	102.0 (93.3-111.8)	97.5 (88.3-104.5)	.137
Pre-operative distalization angle	20.5 (14.3-24.8)	13.0 (7.0-18.0)∗	18.0 (9.8-24.3)	.016
ASES	33.7 (20.9-42.1)	39.4 (27.9-53.1)∗	34.6 (25.5-42.1)	.027
SST	28.7 (16.6-46.7)	26.1 (13.9-46.0)	27.4 (9.1-56.6)	.773
VAS pain	6.2 ± 1.4	6.0 ± 2.0	6.8 ± 1.7	.175
Pre-operative range of motion				
Elevation	105 (74-132)	90 (70-120)	90 (65-110)	.339
Abduction	88 (60-121)	80 (60-100)	80 (60-90)	.602
External rotation	43 (34-60)	30 (20-40)†	30 (15-36)†	<.001
Internal rotation‡	4 (2-4)	2 (2-4)	2 (2-4)	.282

*SST*, Simple Shoulder Test; *VAS*, visual analog scale; *BMI*, body mass index; *COPD*, chronic obstructive pulmonary disease; *SD*, standard deviation; *IQR*, interquartile range; *ASES*, American Shoulder and Elbow Surgeons.

∗ Significant at *P* < .05.

† Significant at *P* < .01.

‡ Internal rotation was scored on a 10-point scale using the following conversions: 2 points = buttock/greater trochanter, 4 points = sacrum-L4, 6 points = L3-L1, 8 points = T12-T8, 10 points = T7-T1.

Pre-operative radiographic assessment revealed similar lateralization angles across groups (*P* = .137). The rotator cuff tear with arthritis cohort demonstrated a lower distalization angle compared with the rotator cuff tear without arthritis cohort (20.5° vs. 13.0°, respectively, *P* = .016). Pre-operative ASES scores were also higher in the rotator cuff tear with arthritis group compared to the rotator cuff tear without arthritis and glenohumeral osteoarthritis cohorts (39.4 vs. 33.7 vs. 34.6, respectively, *P* = .027), although statistically significant, the difference of approximately 5 ASES points did not exceed the established MCID threshold for ASES and therefore is not considered clinically meaningful. External rotation was greater in the rotator cuff tear without arthritis group compared to the rotator cuff tear with arthritis and glenohumeral osteoarthritis cohorts (43°vs. 30°vs. 30°, respectively, *P* < .01), which exceeds the MCID threshold and is therefore considered clinically meaningful.

Perioperative variables did not differ across diagnostic groups as seen in Table II. Subscapularis repair was performed in 14 patients (43.8%) with a rotator cuff tear and absent arthritis, 43 patients (28.7%) with a rotator cuff tear and arthritis, and 17 patients (44.7%) with isolated glenohumeral osteoarthritis. The proportion of subscapularis repair was not different between groups (*P* = .073). Implant selection was similar between groups as well (*P* = .412).

**Table II:** Perioperative characteristics by diagnosis among patients with 2-y follow-up (N = 220).

Variable	Cuff tear without arthritis (N = 32) median (IQR) or n (%)	Cuff tear with arthritis (N = 150) median (IQR) or n (%)	Osteoarthritis (N = 38) median (IQR) or n (%)	*P* value
Subscapularis repair	14 (43.8)	43 (28.7)	17 (44.7)	.073
Implant				.412
32N (10 mm)	2 (6.3)	5 (3.4)	2 (5.6)	
32 -4 (6 mm)	10 (31.3)	46 (30.8)	11 (30.6)	
36N	20 (62.4)	82 (55.0)	16 (44.3)	
36 -4 (2 mm)	0 (0)	5 (3.4)	2 (5.6)	
36 + 4 Restor3D	0 (0)	1 (0.7)	0 (0)	
36 + 6 Biomet	0 (0)	0 (0)	1 (2.8)	
40N (4 mm)	0 (0)	0 (0)	1 (2.8)	
40 -4 (0 mm)	0 (0)	1 (0.7)	0 (0)	
44 + 8	0 (0)	9 (6.0)	3 (8.3)	

*IQR*, interquartile range.

### Post-operative outcomes by diagnosis

All groups demonstrated improvement in ASES scores, SST scores, and VAS from baseline through 2-year follow-up (Table III). ASES scores did not differ across groups at any time point (all *P* > .05). The SST scores at 2 weeks were significantly lower, in the rotator cuff tear without arthritis group compared to the rotator cuff tear with arthritis cohort (8.3 vs. 17.1, respectively, *P* = .033), although not clinically meaningful as it is below the MCID threshold for SST. This difference normalized subsequently by 6-week follow-up (*P* = .370). VAS scores were also similar between all 3 groups at all time points (*P* > .05). Post-operative lateralization and distalization angles were similar between groups as well (*P* = .912 and *P* = .247, respectively).

**Table III:** Post-operative characteristics by diagnosis among patients with 2-y follow-up (N = 221).

Variable	Cuff tear without arthritis (N = 32) median (IQR) or mean ± SD	Cuff tear with arthritis (N = 150) median (IQR) or mean ± SD	Osteoarthritis (N = 39) median (IQR) or mean ± SD	*P* value
Post-operative lateralization angle	83.0 (74.0-86.0)	82.0 (76.0-88.0)	81.0 (73.0-87.0)	.912
Post-operative distalization angle	43.0 (39.5-50.0)	41.0 (36.5-48.0)	38.0 (34.0-46.0)	.247
Post-operative ASES				
2 weeks	50.0 (39.7-59.4)	51.7 (44.7-59.9)	41.9 (34.4-59.2)	.279
6 weeks	59.2 (52.8-73.0)	70.2 (60.1-81.1)	69.6 (53.3-78.6)	.105
3 mo	82.3 (63.2-92.7)	78.8 (67.3-88.0)	77.9 (72.7-88.3)	.756
6 mo	77.7 (61.5-92.6)	85.4 (69.9-92.2)	87.7 (82.3-95.3)	.314
1 y	85.0 (74.2-93.6)	89.3 (79.5-95.3)	87.9 (74.2-97.7)	.527
2 years/most recent	90.3 (85.5-96.0)	89.6 (80.7-95.6)	85.9 (73.1-96.8)	.395
Post-operative SST				
2 weeks	8.3 (8.3-17.1)	17.1 (14.6-35.0)∗	17.1 (14.5-30.1)	.033
6 weeks	45.7 (32.8-74.1)	54.6 (27.0-73.1)	39.9 (23.7-56.7)	.370
3 mo	83.3 (63.6-100)	68.5 (55.7-83.3)	66.7 (45.6-87.5)	.111
6 mo	91.7 (67.5-100)	75.1 (57.2-91.7)	83.1 (65.6-95.8)	.217
1 y	91.7 (65.7-91.7)	83.3. (74.9-91.7)	91.7 (82.9-100)	.188
2 years/most recent	100 (83.3-100)	91.7 (74.5-100)	91.7 (55.2-100)	.080
Post-operative VAS pain				
2 weeks	2.8 ± 2.3	2.5 ± 2.1	3.0 ± 2.5	.654
6 weeks	2.0 ± 2.1	1.6 ± 2.2	1.4 ± 1.8	.383
3 mo	1.3 ± 2.3	1.2 ± 1.6	1.0 ± 1.4	.468
6 mo	1.9 ± 1.7	1.4 ± 2.1	0.9 ± 2.3	.076
1 y	1.2 ± 1.9	0.9 ± 1.8	1.1 ± 1.9	.608
2 years/most recent	0.4 ± 0.8	0.9 ± 1.7	1.2 ± 1.9	.316
SSQ 3 mo	96.4 (87.9-100)	96.4 (87.5-100)	88.4 (81.5-100)	.289
Post-operative range of motion				
Elevation				
10 weeks	130 (125-144)	130 (115-145)	128 (110-140)	.412
6 mo	140 (134-146)	135 (100-144)	120 (83-145)	.343
1 y	140 (130-151)	145 (131-150)	140 (133-154)	.786
2 years/most recent	150 (145-154)	145 (139-155)	155 (130-160)	.498
Abduction				
10 weeks	110 (105-135)	111 (90-130)	105 (90-120)	.278
6 mo	135 (120-135)	120 (90-150)	86 (75-131)	.320
1 y	135 (105-150)	135 (120-150)	135 (113-150)	.712
2 years/most recent	140 (136-158)	140 (120-150)	130 (90-150)	.231
External rotation				
10 weeks	50 (32-56)	39 (30-50)∗	40 (35-55)	.043
6 mo	50 (18-61)	45 (30-50)	53 (38-60)	.313
1 y	41 (38-59)	50 (40-60)	56 (47-66)∗	.041
2 years/most recent	63 (53-70)	50 (40-60)	65 (55-70)†	.014
Internal rotation‡				
10 weeks	2 (2-4)	2 (2-4)	2 (2-4)	.894
6 weeks	2 (2-4)	4 (2-4)	2 (2-4)	.430
1 y	4 (2-6)	4 (2-4)	4 (4-7)	.337
2 years/most recent	4 (4-8)	4 (4-8)	4 (4-8)	.979

*SST*, Simple Shoulder Test; *VAS*, visual analog scale; *SD*, standard deviation; *IQR*, interquartile range; *ASES*, American Shoulder and Elbow Surgeons; *SSQ*, Surgical Satisfaction Questionnaire.

∗ Significant at *P* < .05.

† Significant at *P* < .05.

‡ Internal rotation was scored on a 10-point scale using the following conversions: 2 points = buttock/greater trochanter, 4 points = sacrum-L4, 6 points = L3-L1, 8 points = T12-T8, 10 points = T7-T1.

ROM improved across all domains in all groups. Forward elevation and abduction were comparable between groups at all time points (all *P* > .05). External rotation showed early differences at 10 weeks as the cuff tear without arthritis group demonstrated greater external rotation compared to the rotator cuff tear with arthritis group (50° vs. 39°, respectively, *P* = .043), which exceeds the MCID threshold, representing a clinically meaningful difference. At 1 year, the isolated glenohumeral osteoarthritis group showed greater external rotation compared to the rotator cuff tear without arthritis group (56° vs. 41°, respectively, *P* = .041), exceeding the MCID threshold. At final follow-up, the isolated glenohumeral osteoarthritis group showed greater external rotation compared to the rotator cuff tear with arthritis group (65° vs. 50°, respectively, *P* = .014), exceeding the MCID threshold for external rotation. Internal rotation did not differ at any timepoint (all *P* > .05).

### Post-operative complications by diagnosis

Complications did not differ between groups (*P* = .686) (Table IV). The rotator cuff tear without arthritis group experienced 4 complications (13.8%), consisting of 2 subscapularis tears (6.8%), 1 humerus fracture distal to the stem (3.4%), and 1 infection, which required an incision and drainage with exchange of the poly component (3.4%). The rotator cuff tear with arthritis group had the highest overall complication rate with 24 events (16.9%), primarily acromial fractures (n = 11, 7.7%), followed by 6 subscapularis tears (4.2%), 2 greater tuberosity fractures (1.4%), 2 periprosthetic fractures (1.4%), and 3 other complications including 2 cases of cervical spine pain and 1 patient with heterotopic ossification in the subacromial space (2.1%). The isolated glenohumeral osteoarthritis group demonstrated the lowest complication rate (n = 3, 9.7%), consisting of 1 acromial/scapular fracture, 1 subscapularis tear, and 1 acromial stress reaction (each 3.2%).

**Table IV:** Post-operative complications by diagnosis among patients with 2-y follow-up (N = 202).

Variable	Cuff tear without arthritis (N = 29) n (%)	Cuff tear with arthritis (N = 142) n (%)	Osteoarthritis (N = 31) n (%)	*P* value
Complication (any)	4 (13.8)	24 (16.9)	3 (9.7)	.686
Acromion/scapula fracture	0 (0)	11 (7.7)	1 (3.2)	.330
Humerus fracture (distal to stem)	1 (3.1)	0 (0)	0 (0)	.145
Greater tuberosity fracture	0 (0)	2 (1.4)	0 (0)	1.00
Subscapularis tear	2 (6.9)	6 (4.2)	1 (3.2)	.754
Periprosthetic fracture	0 (0)	2 (1.4)	0 (0)	1.00
Infection	1 (3.4)	0 (0)	0 (0)	.144
Stress reaction	0 (0)	0 (0)	1 (3.2)	.297
Other	0 (0)	3 (2.1)	0 (0)	1.00

### Sub-group analysis: subscapularis repair vs. no repair

Sub-group analysis based on subscapularis repair status was performed to evaluate potential differences in outcomes and complication rates. Seventy-four (33.6%) patients underwent subscapularis repair compared to 146 (66.3%) patients who did not have a subscapularis repair.

Patients undergoing subscapularis repair were younger compared to patients who did not undergo repair (68.8 vs. 73.6, *P* = .001). Patients who underwent subscapularis repair also showed greater pre-operative ROM in all categories: forward elevation (100° vs. 90°, *P* = .003), abduction (90° vs. 75°, *P* = .012), external rotation (40° vs. 30°, *P* < .001), and internal rotation (4 vs. 2, *P* = .004). Other demographics and baseline characteristics were similar between groups (*P* > .05 for all) as seen in Table V. Implant selection also varied between the subscapularis repair and no repair groups (*P* < .001). Although the 36N sized glenosphere remained the most common in both groups (repair = 43.1%, no repair 60.1%) as seen in Table VI.

**Table V:** Pre-operative characteristics by intervention among patients with 2-y follow-up (N = 220).

Variable	Subscapularis repair (N = 74) median (IQR), mean ± SD, or n (%)	No subscapularis repair (N = 146) median (IQR), mean ± SD, or n (%)	*P* value
Age (years)	68.8 (65.0-74.5)	73.6 (66.8-78.7)	.001
BMI (kg/m^2^)	28.3 (24.0-34.3)	29.5 (26.3-36.8)	.153
Female sex	37 (50.0)	89 (61.0)	.121
Surgical side			.344
Right	45 (60.8)	79 (54.1)	
Left	29 (39.2)	67 (45.9)	
Surgery on dominant arm	37 (61.7)	62 (53.0)	.271
Prior shoulder surgery	20 (27.0)	30 (21.0)	.316
Diagnosis			.111
Cuff tear	57 (77.0)	125 (85.6)	
Osteoarthritis	17 (23.0)	21 (14.4)	
Arthritis	60 (81.1)	128 (87.7)	.190
Comorbidities			
Osteopenia	20 (27.0)	36 (25.0)	.746
COPD	2 (2.7)	8 (5.6)	.500
Autoimmune disorder	3 (4.1)	12 (8.4)	.274
Diabetes	8 (13.3)	18 (15.4)	.715
Current smoker	3 (5.1)	2 (1.8)	.339
Anticoagulant	13 (21.7)	30 (25.6)	.559
Walch classification			.949
A1	58 (79.5)	115 (78.8)	
A2	3 (4.1)	6 (4.1)	
B1	0 (0)	1 (0.7)	
B2	5 (6.8)	6 (4.1)	
B3	7 (9.6)	16 (11.0)	
C	0 (0)	1 (0.7)	
D	0 (0)	1 (0.7)	
Pre-operative lateralization angle	105.5 (96.3-110.8)	100.0 (89.0-108.3)	.176
Pre-operative distalization angle	17 (9.5-24.0)	14.0 (7.0-17.0)	.059
ASES	39.1 (30.6-46.9)	37.0 (24.5-51.2)	.669
SST	28.9 (14.8-53.1)	25.1 (11.8-46.2)	.298
VAS pain	6.1 ± 1.8	6.2 ± 2.0	.652
Pre-operative range of motion			
Elevation	100 (80-130)	90 (65-110)	.003
Abduction	90 (70-100)	75 (60-100)	.012
External rotation	40 (30-53)	30 (20-40)	<.001
Internal rotation∗	4 (2-4)	2 (2-4)	.004

*SST*, Simple Shoulder Test; *VAS*, visual analog scale; *BMI*, body mass index; *COPD*, chronic obstructive pulmonary disease; *SD*, standard deviation; *IQR*, interquartile range; *ASES*, American Shoulder and Elbow Surgeons.

∗ Internal rotation was scored on a 10-point scale using the following conversions: 2 points = buttock/greater trochanter, 4 points = sacrum-L4, 6 points = L3-L1, 8 points = T12-T8, 10 points = T7-T1.

**Table VI:** Perioperative characteristics by intervention among patients with 2-y follow-up (N = 217).

Variable	Subscapularis repair (N = 74) median (IQR) or n (%)	No subscapularis repair (N = 143) median (IQR) or n (%)	*P* value
Implant			<.001
32N (10 mm)	9 (12.2)	0 (0)	
32 -4 (6 mm)	29 (39.1)	38 (26.6)	
36N	32 (43.1)	86 (60.1)	
36 -4 (2 mm)	1 (1.4)	6 (4.2)	
36 + 4 Restor3D	0 (0)	1 (0.7)	
36 + 6 Biomet	1 (1.4)	0 (0)	
40N (4 mm)	1 (1.4)	0 (0)	
40 -4 (0 mm)	0 (0)	1 (0.7)	
44 + 8	1 (1.4)	11 (7.7)	

*IQR*, interquartile range.

Functional outcomes improved in both groups regardless of subscapularis repair (Table VII). The repair group demonstrated early improvements in SST and external rotation compared to the no-repair group. However, at final follow-up, there were no differences in ASES (*P* = .172), SST (*P* = .218), VAS pain (*P* = .401), or ROM outcomes between the intervention groups (*P* > .05 for all). The group showed a greater distalization angle compared to the no repair group (44° (40-48) vs. 40° (34-47), *P* = .040). The subscapularis repair group showed a greater rate of complications compared to no repair (n = 15 (22.7%) vs. n = 16 (11.8%), *P* = .043) as seen in Table VIII. Of the complications in the repair group, the majority were due to subscapularis tear (n = 9, 13.6%). In the no repair group, the majority of complications were due to acromion/scapula fractures (n = 10, 7.4%).

**Table VII:** Post-operative characteristics by intervention among patients with 2-y follow-up (N = 220).

Variable	Subscapularis repair (N = 74) median (IQR) or mean ± SD	No subscapularis repair (N = 146) median (IQR) or mean ± SD	*P* value
Post-operative lateralization angle	81.0 (75.0-86.0)	83.0 (76.0-88.0)	.373
Post-operative distalization angle	44.0 (40.0-48.0)	40.0 (34.0-47.0)	.040
Post-operative ASES			
2 weeks	47.3 (39.5-57.1)	51.7 (42.1-61.1)	.273
6 weeks	65.3 (50.8-77.0)	71.6 (60.7-80.3)	.086
3 mo	77.8 (64.8-89.6)	79.0 (68.5-88.1)	.707
6 mo	84.5 (70.2-92.8)	85.8 (68.4-92.2)	.809
1 y	86.0 (75.3-94.6)	89.6 (79.9-95.5)	.313
2 years/most recent	88.5 (78.1-95.8)	89.4 (80.4-95.7)	.729
Post-operative SST			
2 weeks	8.3 (0-25.0)	17.6 (17.1-35.0)	<.001
6 weeks	52.4 (25.7-64.9)	49.9 (28.5-74.1)	.568
3 mo	75.9 (52.4-91.7)	68.7 (55.0-83.3)	.675
6 mo	83.1 (64.2-91.7)	75.0 (57.3-91.7)	.179
1 y	91.7 (74.5-100)	83.3 (74.5-91.7)	.504
2 years/most recent	91.7 (74.8-100)	90.8 (74.9-100)	.409
Post-operative VAS pain			
2 weeks	3.0 ± 2.2	2.4 ± 2.2	.161
6 weeks	2.0 ± 2.3	1.4 ± 2.0	.127
3 mo	1.3 ± 1.9	1.1 ± 1.6	.552
6 mo	1.3 ± 1.8	1.4 ± 2.2	.798
1 y	1.3 ± 2.0	0.8 ± 1.7	.043
2 years/most recent	1.3 ± 2.1	0.7 ± 1.4	.078
SSQ 3 mo	96.4 (81.3-100)	95.8 (87.5-100)	.956
Post-operative range of motion			
Elevation			
10 weeks	135 (112-135)	130 (115-141)	.138
6 mo	140 (103-149)	134 (100-143)	.180
1 y	145 (135-151)	140 (130-150)	.262
2 years/most recent	145 (140-155)	145 (137-160)	.938
Abduction			
10 weeks	120 (90-130)	105 (90-130)	.315
6 mo	129 (92-149)	120 (88-135)	.305
1 y	149 (127-150)	135 (105-150)	.053
2 years/most recent	140 (115-150)	140 (120-158)	.526
External rotation			
10 weeks	45 (32-55)	39 (30-50)	.064
6 mo	56 (48-62)	45 (30-50)	.001
1 y	55 (44-62)	47 (37-60)	.005
2 years/most recent	60 (43-65)	53 (40-60)	.166
Internal rotation∗			
10 weeks	2 (2-4)	2 (2-4)	.773
6 weeks	2 (2-4)	4 (2-4)	.488
1 y	4 (2-8)	4 (2-4)	.136
2 years/most recent	4 (4-8)	4 (4-6)	.305

*SST*, Simple Shoulder Test; *VAS*, visual analog scale; *SD*, standard deviation; *IQR*, interquartile range; *ASES*, American Shoulder and Elbow Surgeons; *SSQ*, Surgical Satisfaction Questionnaire.

∗ Internal rotation was scored on a 10-point scale using the following conversions: 2 points = buttock/greater trochanter, 4 points = sacrum-L4, 6 points = L3-L1, 8 points = T12-T8, 10 points = T7-T1.

**Table VIII:** Post-operative complications by intervention among patients with 2-y follow-up (N = 202).

Variable	Subscapularis repair (N = 66) n (%)	No subscapularis repair (N = 136) n (%)	*P* value
Complication (any)	15 (22.7)	16 (11.8)	.043
Acromion/scapula fracture	2 (3.0)	10 (7.4)	.344
Humerus fracture (distal to stem)	0 (0)	1 (0.7)	1.00
Greater tuberosity fracture	0 (0)	2 (1.5)	1.00
Subscapularis tear	9 (13.6)	0 (0)	<.001
Periprosthetic fracture	1 (1.5)	1 (0.7)	.548
Infection	1 (1.5)	0 (0)	.327
Stress reaction	0 (0)	1 (0.7)	1.00
Other	2 (3.0)	1 (0.7)	.249

## Discussion

The primary finding of this study is that patients undergoing rTSA for irreparable rotator cuff tear without arthritis, rotator cuff tear with arthritis, or primary glenohumeral osteoarthritis demonstrated comparable outcomes at 2-year follow-up. Although early post-operative variations in SST and external rotation were present between rotator cuff tears with arthritis compared to rotator cuff tears without arthritis, these differences resolved before final follow-up. Early differences in external rotation may have been driven in part by greater pre-operative external rotation in the rotator cuff tear without arthritis cohort, leading to variability in early post-operative recovery but diminishing over time. All 3 groups experienced significant improvements in ASES, SST, VAS pain scores, and ROM. This suggests that rTSA is a reproducible and successful treatment option for glenohumeral osteoarthritis, irreparable rotator cuffs without arthritis, and rotator cuff tears with arthritis when performed uniformly with a single implant system.

External rotation was the only outcome that demonstrated persistent differences between diagnostic groups. At 1 year, patients with primary glenohumeral osteoarthritis demonstrated 15° greater external rotation than patients with irreparable rotator cuff tears without arthritis, and at final follow-up, patients with primary glenohumeral osteoarthritis demonstrated 15° greater external rotation than patients with rotator cuff tear arthropathy. These differences exceeded the established MCID threshold for external rotation. This may be due to the intact posterosuperior rotator cuff in osteoarthritis patients, allowing for greater external ROM. However, patients with rotator cuff tears without arthritis had significantly greater external rotation pre-operatively. Waterman et al^28^ also found patients with glenohumeral osteoarthritis and intact rotator cuffs showed greater external rotation after rTSA compared to rotator cuff tear arthropathy. Saini et al^20^ found similar results as well and hypothesized the intact rotator cuff in glenohumeral osteoarthritis patients allows for balanced force coupling and greater dynamic stability compared to patients with rotator cuff arthropathy, leading to greater improvements in ROM. This may also be due to smaller cohort sizes, particularly for glenohumeral osteoarthritis and rotator cuff tears without arthritis, leading to greater variability in data.

Our results differ from many other observational studies comparing multiple pre-operative diagnoses. Testa et al^24^ performed a retrospective study of 625 patients from 2 institutional databases that showed rTSA for glenohumeral osteoarthritis showed significantly superior post-operative VAS pain scores, ASES scores, and Single Assessment Numeric Evaluation scores, forward elevation, internal rotation, and external rotation compared to rotator cuff tear arthropathy and massive rotator cuff tears without arthritis. They also found that patients undergoing rTSA for glenohumeral osteoarthritis showed the greatest rates of reaching the MCID for ASES scores at 94.5% and substantial clinical benefit (SCB) at 87.2%, which were significantly greater than the rotator cuff tear arthropathy (MCID: 87.2%, *P* = .006; SCB: 75.6%, *P* < .001) and massive rotator cuff tear without arthritis (MCID: 92.3%, *P* = .290, SCB: 74.4%, *P* < .001).^24^ Puzzitiello et al^19^ also compared cohorts of patients undergoing rTSA glenohumeral osteoarthritis and rotator cuff tear arthropathy, as well as aTSA for glenohumeral osteoarthritis. They found that patients undergoing rTSA and aTSA for glenohumeral osteoarthritis reached MCID for ASES scores earlier than rTSA for rotator cuff tear arthropathy and at 2-years a significantly higher percentage of rTSA for glenohumeral osteoarthritis patients achieved MCID and SCB compared to rTSA for rotator cuff tear arthropathy (MCID: 100% vs. 95.5%, *P* = .003, SCB: 94.6% vs. 86.4%, *P* = .036).^19^

Similarly, Saini et al^20^ compared patients undergoing rTSA for glenohumeral osteoarthritis to rotator cuff tear arthropathy and found that glenohumeral osteoarthritis patients had greater post-operative forward elevation, internal rotation, and external rotation (*P* < .01), and a significantly greater amount of patients reached MCID and SCB for ASES scores at 2 years compared to rotator cuff tear arthropathy (MCID: 97.5% vs. 86.7%, *P* < .001; SCB: 90.4% vs. 71.7%, *P* < .01). Forlizzi et al^9^ also found that pre-operative glenohumeral osteoarthritis was the strongest predictor of excellent post-operative outcomes defined as a top quartile of ASES score (odds ratio: 5.6, 95% CI: 1.2-26.5, *P* = .03). Nové-Josserand et al^17^ also found that patients undergoing rTSA for glenohumeral osteoarthritis with an intact cuff showed improved post-operative Constant Murley scores (73 ± 14.3) compared to patients with glenohumeral osteoarthritis with a torn cuff (66.1 ± 14.6, *P* < .001) and secondary osteoarthritis defined by the Hamada et al^11^ classification (64.1 ± 14.8, *P* < .001). Lindbloom et al^14^ found that rTSA provided improvement regardless of pre-operative indication which was similar to our study, though they did find greater pre-operative to post-operative improvement in ASES and VAS (*P* < .01 and *P* < .05) in patients undergoing rTSA for glenohumeral arthritis with an intact cuff compared to rotator cuff tear arthropathy, massive rotator cuff tears with and without arthritis, proximal humerus fractures, malunion, non-union, and inflammatory arthropathies. Our data do not align with these studies, as no group performed significantly better than another. These discrepancies may relate to the single-institution design of our study, consistent implant selection, and standardized post-operative management, which may reduce variability between diagnostic groups. However, our study included a smaller cohort than these prior studies, which may have limited the statistical power to detect differences between diagnostic groups, particularly for lower frequency outcomes such as complications.

While the majority of studies have shown different outcomes for various pre-operative diagnoses, Kim et al^13^ did show similar results to our study, as they found no significant difference in ROM, strength, VAS, Constant Murley, or University of California Los Angeles (UCLA) scores between those with rotator cuff tear arthropathy compared to irreparable rotator cuff tears. Additionally, no significant differences in these same outcome measures were observed when comparing rotator cuff tear arthropathy and irreparable rotator cuff tears to arthritic pathologies, including glenohumeral osteoarthritis, rheumatoid arthritis, and posttraumatic arthritis.^13^

Multiple systematic reviews have evaluated outcomes following rTSA across pre-operative diagnostic groups. Kennedy et al^12^ reported that rTSA provides reliable functional improvement regardless of pre-operative diagnosis and demonstrated similar outcomes among patients undergoing rTSA for glenohumeral osteoarthritis, rotator cuff tear arthropathy, and irreparable rotator cuff tears. These findings are consistent with the results of this study. Inferior functional outcomes and higher complication rates were primarily observed in revision arthroplasty, proximal humerus fractures, and rheumatoid arthritis.^12^ These diagnostic groups are well known to have worse outcomes and were excluded from this analysis. Kennedy et al^12^ also highlighted that much of the existing literature consists of level IV retrospective case series with a high risk of bias, underscoring the need for further investigation. Similarly, Coscia et al^8^ also performed a systematic review and found that patients with primary osteoarthritis, massive rotator cuff tears with arthritis, and massive rotator cuff tears without arthritis all performed reliably after rTSA, and complex proximal humerus fractures, fracture sequelae, and revision rTSA showed less consistent results. This supports our findings of comparable outcomes for osteoarthritis and rotator cuff tears with and without arthritis.

The complication rate in our cohort was favorable, with no significant difference between groups. The rotator cuff tear with arthritis group had the highest complication rate at 16.8%, followed by the rotator cuff tear without arthritis (13.8%) and osteoarthritis (9.7%). This differs from Testa et al,^24^ who found significantly lower complication rates in patients with osteoarthritis compared to rotator cuff tear arthropathy (3.9% vs. 9.1%, *P* = .024). The systematic review by Kennedy et al^12^ showed similar rates of complication (rotator cuff tear arthropathy = 7.4%, rotator cuff tear without arthritis = 20%, and glenohumeral osteoarthritis 1.4%), though the variation between diagnostic groups differs from our results. The complication rates of this study are also lower than previously reported on 10-year outcomes following rTSA, which showed complication rates up to 36%.^3^ However, the lack of significant findings may again be due to smaller cohort sizes, which limits the statistical power to detect significant differences.

Sub-group analysis of subscapularis repair revealed that while patients undergoing repair were younger and had greater pre-operative ROM, long-term functional outcomes at two years were equivalent to those without repair. This aligns with a previous study by Vourazeris et al,^27^ who found no significant differences in functional, ROM, or patient-reported outcomes. Friedman et al^10^ found statistical differences in the improvement of SST and Constant Murley scores as well as internal and external rotation between repair and tenotomy; however, they state these differences were unlikely to meet clinical significance. Subscapularis repair remains a topic of debate, in addition to determining if it results in significantly different outcomes compared to tenotomy.^2^^,^^7^ However, this sub-group analysis is inherently limited by selection bias, as surgical decision-making was not randomized and was not controlled for baseline subscapularis integrity or technique.

In this study, subscapularis repair was associated with a significantly higher overall complication rate, largely due to tendon failure. However, tendon failure is a unique complication to the repair group, and when looking at other complications, the repair group showed an 8.1% complication rate (6/74) compared to the 11.8% (16/143) complication rate in the non-repair group. Acromial or scapular fractures occurred more frequently in the non-repair group (7.4%, n = 10) than in the repair group (3.0%, n = 2), although this difference did not reach statistical significance (*P* = .334). When excluding tendon failure, our results align with previous studies that have shown similar complication rates between repair and tenotomy, especially with a lateralized implant.^2^^,^^7^^,^^10^^,^^27^

The lack of significant differences in baseline demographics and risk factors, including age, sex, BMI, and comorbidities, including osteopenia, COPD, autoimmune disease, diabetes mellitus, smoking status, and anticoagulant use, is a strength of this study. These risk factors have previously been associated with inferior outcomes following rTSA.^5^^,^^9^^,^^25^ Psychosocial factors, including anxiety, depression, opioid use, and workers' compensation, have also been associated with inferior post-operative outcomes and increased complications.^6^^,^^26^ Disease progression and severity may also play a role in patient outcomes and satisfaction as well.^21^ The similar demographics across cohorts, as well as consistent implants, surgical technique, and follow-up protocol in this study helps isolate the effect of pre-operative diagnosis on post-operative outcomes. Additionally, the minimum 2-year follow-up, with a high proportion of patients completing the full follow-up period (91.8%), strengthens the validity of these findings, as most early complications and functional improvements following rTSA occur within this timeframe.

However, this study has several limitations. The retrospective design of the study introduces the potential for selection bias and limits control over confounding variables that may influence post-operative outcomes. Although patient-reported outcomes were largely complete at 2-year follow-up, minor missing data resulted in small variations in sample size across analyses. Complication data were available for a smaller subset of shoulders, with 19 shoulders lacking complication data, which may introduce bias in the estimated complication rates. This study is also limited by its relatively small and uneven cohort sizes compared with prior studies evaluating outcomes across rTSA indications. Consequently, the study may have been underpowered to detect small differences in outcomes. Also, this is a single surgeon study using a single implant system and a standardized perioperative protocol, which may limit the generalizability of our findings to other surgeons using different implants or rehabilitation protocols. An additional limitation is that 1 author serves as a consultant to and receives royalties from Enovis, the manufacturer of the implant system used in this study. Although data collection, analysis, and interpretation were conducted according to institutional research protocols, this relationship represents a potential source of bias. Clinical outcomes were evaluated at a minimum 2-year follow-up, which limits the ability to draw long-term conclusions as well. Larger, multicenter, and prospective studies are warranted to assess outcomes over longer follow-up periods and across different surgical techniques and implants.

## Conclusion

At 2-year follow-up, rTSA provided comparable improvements in pain, function, and patient-reported outcomes for patients treated for irreparable rotator cuff tears without arthritis, rotator cuff tear arthropathy, and isolated glenohumeral osteoarthritis. Complication rates were low and not significantly affected by the pre-operative diagnosis. Radiographic outcomes were also similar between groups. Subscapularis repair was associated with a higher complication rate, primarily due to failure of the repaired tendon. These findings support the generalizability and consistency of rTSA outcomes across common pre-operative indications.

## Disclaimers:

Funding: No funding was disclosed by the authors.

Conflicts of interest: Brian L. Badman is a consultant and receives royalties from Enovis. Any additional authors, their immediate families, and any research foundations with which they are affiliated have not received any financial payments or other benefits from any commercial entity related to the subject of this article.
